# Comparative Analysis Delineates the Transcriptional Resistance Mechanisms for Pod Borer Resistance in the Pigeonpea Wild Relative *Cajanus scarabaeoides* (L.) Thouars

**DOI:** 10.3390/ijms22010309

**Published:** 2020-12-30

**Authors:** Isaac Njaci, Abigail Ngugi-Dawit, Richard O. Oduor, Leah Kago, Brett Williams, Linh Thi My Hoang, Sagadevan G. Mundree, Sita R. Ghimire

**Affiliations:** 1 Biosciences Eastern and Central Africa—International Livestock Research Institute (BecA-ILRI) Hub, Nairobi P.O. Box 30709-00100, Kenya; injaci@cgiar.org (I.N.); liakagoh@gmail.com (L.K.); 2 Centre for Agriculture and the Bioeconomy (CAB), Queensland University of Technology, Brisbane City, QLD 4000, Australia; a.dawit@hdr.qut.edu.au (A.N.-D.); b.williams@qut.edu.au (B.W.); lt.hoang@qut.edu.au (L.T.M.H.); 3 Department of Biochemistry, Microbiology and Biotechnology, Kenyatta University, Nairobi P.O. Box 43844-00100, Kenya; oduor.richard@ku.ac.ke

**Keywords:** pigeonpea, crop wild relatives, *Helicoverpa armigera*, pod borer, insect resistance, transcriptomics, *Cajanus scarabaeoides*

## Abstract

Insect pests pose a serious threat to global food production. Pod borer (*Helicoverpa armigera* (Hübner)) is one of the most destructive pests of leguminous crops. The use of host resistance has been an effective, environmentally friendly and sustainable approach for controlling several agricultural pests. The exploitation of natural variations in crop wild relatives could yield pest-resistant crop varieties. In this study, we used a high-throughput transcriptome profiling approach to investigate the defense mechanisms of susceptible cultivated and tolerant wild pigeonpea genotypes against *H. armigera* infestation. The wild genotype displayed elevated pest-induced gene expression, including the enhanced induction of phytohormone and calcium/calmodulin signaling, transcription factors, plant volatiles and secondary metabolite genes compared to the cultivated control. The biosynthetic and regulatory processes associated with flavonoids, terpenes and glucosinolate secondary metabolites showed higher accumulations in the wild genotype, suggesting the existence of distinct tolerance mechanisms. This study provides insights into the molecular mechanisms underlying insect resistance in the wild pigeonpea genotype. This information highlights the indispensable role of crop wild relatives as a source of crucial genetic resources that could be important in devising strategies for crop improvement with enhanced pest resistance.

## 1. Introduction

Pigeonpea (*Cajanus cajan* (L.) Mill sp.) is the sixth most important grain legume in the world. It is predominantly grown in Asia, Africa and the Caribbean islands. India is the largest producer, with 4.29 million tons, accounting for 72% of the world’s pigeonpea production [1]. The crop plays a vital role in rainfed agriculture to sustain the livelihood of millions of people in semi-arid tropics and subtropics. It is an important legume that not only provides food grains, forage, domestic energy source and herbal medicine but, also, improves soil fertility by fixing atmospheric nitrogen and reducing soil erosion [2]. Pigeonpea productivity has stagnated due to abiotic and biotic stresses, inferior crop varieties and inefficient management practices. However, biotic stress from insect pests comprises the major production constraint [3]. The pod borer (*Helicoverpa armigera* (Hübner)) is the most damaging pest of pigeonpea. It is a peculiar pest due to its high mobility, diverse plant host range, high reproductive rates and diapause [4]. Despite the use of over US$ one billion worth of insecticides, *H. armigera* accounts for an estimated US$ 317 million of annual crops loss in the semi-arid tropics [5] and over US$ two billion globally [6].

The greatest potential in integrated pest management for reduced yield losses, especially in developing countries, lies in the development of insect-resistant cultivars [7]. The screening of over 14,000 pigeonpea accessions revealed low-to-moderate levels of resistance against *H. armigera* [8]. However, pigeonpea wild relatives have shown high levels of resistance to *H. armigera* [5,9]. The two most *H. armigera*-resistant wild pigeonpea species are *Cajanus scarabaeoides* and *Cajanus cinereus* [5,10]. Despite their poor agronomic characteristics, the pigeonpea wild relatives possess great potential as a source of new genes and markers for enhancing the resistance to biotic stress based on their high genetic diversity, including pest and disease resistance traits [11,12]. Numerous strategies, including metabolomics and transcriptomics, have been initiated to utilize the genetic resources of crop wild relatives (CWRs) for the improvement of various crop varieties [13]. Genomic approaches have been widely used to identify genes or genomic regions controlling complex traits, while high-throughput sequencing has offered opportunities to efficiently discover single nucleotide polymorphism (SNPs) associated with important traits [14,15].

Plants are frequently attacked by insects and pathogens. Their sessile nature enables them to evolve elaborate direct and indirect defense mechanisms against these biotic stresses [16]. Plant defenses are determined not only by biochemical and morphological features but, also, by transduction and interaction via signaling molecules. In addition to the physical barriers, including trichomes and thick cell walls, plants initiate defense mechanisms by mobilizing defense signaling pathways for the activation of defense genes and the synthesis of secondary metabolites and toxic compounds such as terpenoids, alkaloids, anthocyanins, phenols and quinones that either eliminate or retard the development of the pest [17,18]. Several phytohormones, including jasmonic acid (JA), salicylic acid (SA), ethylene (ET), abscisic acid (ABA), brassinosteroids (BR) and gibberellin (GA), regulate the plant defense system [19,20]. The plants also indirectly defend themselves through the release of volatile substances that attract the natural enemies of the invading pests [21]. Plants actively respond to herbivory by inducing various defense mechanisms in both damaged (locally) and nondamaged tissues (systemically). The response to herbivory is activated through the recognition of herbivory-induced damage. [22]. The herbivory-induced changes are mediated by signaling networks, including receptors/sensors, Ca^2+^ influx pathways, kinase cascades, reactive oxygen species formation (ROS), secondary metabolites, mitogen-activated protein kinases (MAPKs), calcium-dependent protein kinases (CDPKs) and phytohormone signaling pathways [18,23]. Despite the major strides in elucidating the pest-plant interactions, the underlying insect-host interaction mechanisms are still poorly understood.

The development of resistant crop varieties is bearing fruit; however, pathogen and insect pests evolve rapidly. To mitigate this evolutionary arms race, efforts have been focused on exotic genetic resources such as CWRs to develop biotic stress-resistant varieties using various strategies, including transcriptomics. Although high-throughput RNA-Seq has been used to analyze the herbivory-associated genes expression profiles in crops such as rice [24] and the common bean [25], no such approaches on pigeonpea wild relatives have been reported. In this study, a medium duration indeterminate and *H. armigera* cultivated susceptible pigeonpea KAT 60/8 (CT) genotype and a resistant *Cajanus scarabaeoides* wild relative accession (WT) were used to explore the untapped genetic resources associated with *H. armigera* herbivory resistance. Transcriptomic responses between the WT and CT genotypes 24 h postinfestation were assessed using RNA-Seq. The key genes, signaling pathways and metabolites utilized by the wild pigeonpea in defense against *H. armigera* infestation were identified. The results indicate that wild pigeonpea relatives use superior defense mechanisms. Once known, these strategies can be applied for crop improvement and the development of *H. armigera*-resistant pigeonpea varieties.

## 2. Results

### 2.1. Wild Pigeonpea Cajanus scarabaeoides Is Resistant to Helicoverpa armigera Herbivory

**Figure 1:**
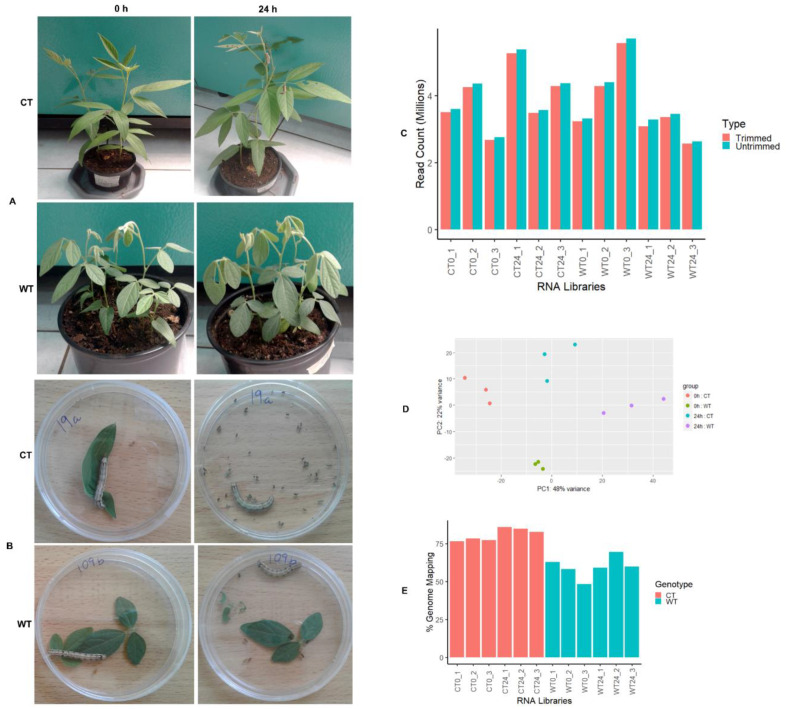
*Helicoverpa armigera* bioassays and high-throughput sequencing of cultivated pigeonpea (KAT 60/8) and wild relative (*Cajanus scarabaeoides*). (**A**) Whole plant bioassay and (**B**) detached leaves bioassay. (**C**) Reads distribution statistics of sequenced libraries. (**D**) Principal component analysis (PCA) plot showing the clustering of sequenced libraries, and (**E**) percentage reads mapping of cultivated and wild relative genotypes against the pigeonpea genome.

*Helicoverpa armigera* is highly adapted to feeding on various plant parts. However, damage to the reproductive parts, particularly the flowers and developing seeds, results in direct yield and economic losses. To investigate the genotype-specific response to the pod borer infestation, we conducted an insect bioassay on the cultivated susceptible cultivar KAT 60/8 (CT) [26] and a resistant wild relative accession (WT) [5] using the whole plant and detached leaves assays. Four-week-old seedlings and detached leaves were subjected to *H. armigera* infestation for a period of 24 h. The results show severe defoliation in CT, while minimal leaf damage was observed on the wild relative seedlings under the whole plant and petri plate assays (Figure 1A,B).

### 2.2. Generation of Transcriptomes from Susceptible and Resistant Pigeonpea Accessions

Observation of the leaf damage from the insect bioassay experiment showed that KAT 60/8 (CT) was susceptible, while the wild relative *Cajanus scarabaeoides* (WT) was tolerant to *H. armigera* infestation Figure 1A,B). To further investigate the mechanisms underlying the genotype-specific insect damage resistance, we performed high-throughput sequencing of the two genotypes under *H. armigera* infestation. Four CT and WT triplicate RNA libraries were sequenced using an Illumina Mi-Seq platform. A total of 23 million high-quality paired-end reads averaging 1.3–2.8 million reads per library were generated (Figure 1C,D). Preprocessed reads were mapped against the pigeonpea reference genome (C. cajan_V1.0) using a Tophat aligner. The overall reads mapping rate in CT was 77% (0 h) and 84% (24 h), while the WT had lower reads mapping rates of 63% (0 h) and 56% (24 h), respectively. The reads mapping resulted in the identification of 31,841 nonredundant unigenes.

### 2.3. Herbivory-Induced Transcriptome Changes between Susceptible Cultivated and Tolerant Wild Pigeonpea Genotypes

**Figure 2:**
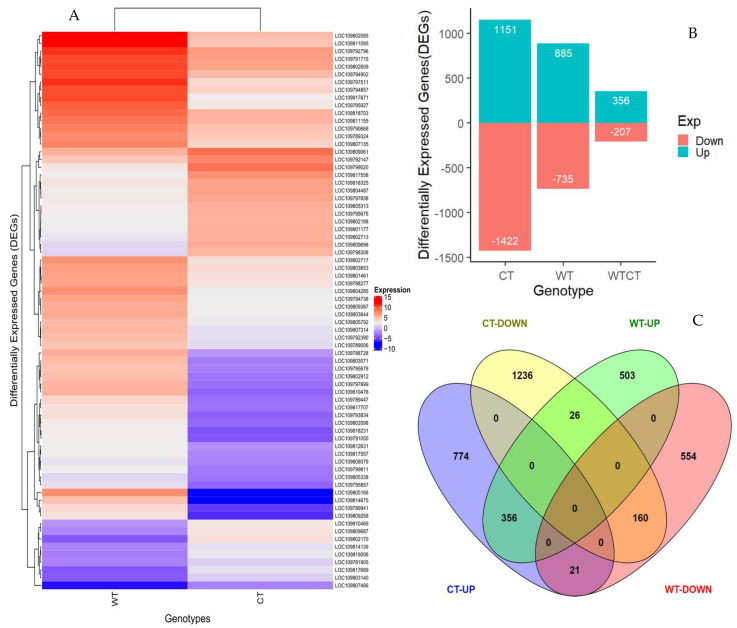
Differentially expressed genes in susceptible cultivated (CT) and resistant wild (WT) pigeonpea genotypes upon pod borer infestation. (**A**) Heatmap showing differentially expressed genes between CT and WT after 24 h of pod borer infestation. (**B**) Bar graph showing the disparate expression of genotype-specific and shared genes between CT and WT. (**C**) Cross-comparison Venn diagram showing the number of differentially expressed genes in CT and WT.

The raw reads counts were converted into counts per million (CPM), and the log transformed CPM (log-CPM) values were used for the expression level analysis. The unigenes with an expression of more than two-fold change, a *p*-value < 0.001 and false discovery rate (FDR) < 0.05 were considered differentially expressed. Distinct expression responses between the accessions were observed upon insect infestation, and a total of 3630 (11.4%) unigenes were differentially expressed (Figure 2A). In CT, 2573 unigenes were differentially expressed, with 1151 and 1422 genes significantly up- and downregulated, respectively (Figure 2B and Supplementary Table S1). The resistant WT had 1620 differentially expressed unigenes comprising 885 up- and 735 downregulated genes (Figure 2B and Supplementary Table S2). Among the differentially expressed genes (DEGs), 563 (15.5%) unigenes were shared between the CT and WT genotypes (Figure 2B,C and Supplementary Table S3). However, 1057 genes, comprising 503 up- and 554 downregulated, were unique to WT, while 2010 unigenes, 774 up- and 1236 down-regulated, were specific to the CT genotype (Figure 2C). The WT-specific DEGs and those with enhanced expression changes in the WT could be attributed to regulatory roles and cellular responses in defense against *H. armigera* infestation.

### 2.4. Plant Hormones and Their Role in Plant Defense during Insect Herbivory

Hormone signaling plays a key role in plant immunity. The plant defense system is regulated by a suite of phytohormones. In this study, several DEGs were associated with biosynthesis and a response to jasmonic acid (JA), ethylene (ET), salicylic acid, abscisic acid, cytokinin and gibberellic acid (GA). The key genes that showed an enhanced expression in the JA biosynthesis pathway included lipoxygenases (XM_020355903.2 and XM_020367824.2), oxophytodienoate reductase (XM_020370652.1), allene oxide cyclase (XM_020348845.1), allene oxide synthase (XM_020361875.1) and jasmonate O-methyltransferase (XM_020365833.2). Similarly, ethylene biosynthesis pathway enzymes 2-oxoglutarate (XM_020377202.1, XM_020348803.2), aminocyclopropane-1-carboxylate synthase (XM_020361446.1, XM_020367997.1) and 1-aminocyclopropane-1-carboxylate oxidase (XM_020368452.1) showed enhanced induction. The cytokinin gene encoding zeatin O-glucosyltransferase-like (XM_020357438.1) was upregulated. However, in contrast to JA and ET, the GA pathway-associated enzymes gibberellin oxidase (XM_020372878.2) and the gibberellin-regulated protein (XM_020347175.2) were downregulated (Table 1 and Supplementary Table S3). When the expression of the hormone-related enzymes was compared between the two accessions, their expression in response to insect herbivory was higher in the WT. The expression of two cytokinin pathway genes: cytokinin hydroxylase-like (XM_020353072.1) catalyzing the biosynthesis of trans-zeatin and cytokinin dehydrogenase 3-like (XM_020370055.1) involved in the oxidation of cytokinin was enhanced in the WT, with no evident expression in the CT (Supplementary Table S3).

### 2.5. Transcription Factors Associated with H. armigera Herbivory

Several transcription factors (TFs) are associated with plant defense responses and regulations. In this study, the TFs WRKY, MYB, basic helix-loop-helix leucine zipper (bHLH), Ethylene-Responsive (ERF), NAC (NAM, ATAF1/2 and CUC2)-domain and bZIP showed differential expressions in response to the pod borer infestation. In CT, WRKY57, WRKY30, WRKY41, WRKY51, WRKY69 and WRKY24 were upregulated, while the expression of WRKY71, WRKY44 and WRKY49 was suppressed. Seven were WT-unique; WRKY51, WRKY75, WRKY41, WRKY28, WRKY31, WRKY23 and WRKY22 were significantly induced upon infestation (Table 1 and Supplementary Table S3). The large number of WRKY TFs suggests an important role of these TFs in response to *H. armigera* infestation. Two basic helix-loop-helix (bHLH), jasmonate-associated MYC2 (XM_020349871.1) and MYC4 (XM_020351385.1) TFs controlling JA biosynthesis were significantly induced in both CT and WT (Table 1). Unique members of the ethylene-responsive transcription factors showed a differential expression between the genotypes. In CT, ERF017, ERF1A and RAP2-7-like (XM_020383974.1) were upregulated, while ERF-WIN1-like (XM_020350358.1) and AP2-like-ERF (XM_020374031.1) were downregulated. The TFs ERF-1-like, ERF110, ERF113 and ERF RAP2-6-like (XM_020357051.1) were upregulated in the WT genotype (Supplementary Table S3). However, ethylene-responsive transcription factor ABR1-like (XM_020349666.1) and ERF1B-like (XM_020382274.1) were significantly expressed in both genotypes. In the WT, the expression of bZIP transcription factor 11-like (XM_020382733.1) was highly enhanced, while its expression in the CT was suppressed (Table 1).

### 2.6. Calcium/Calmodulin-Mediated Signaling in H. armigera Resistance

Calcium (Ca^2+^) is a universal second messenger in signal transduction and a mediator in many biological processes associated with growth, development and physiological responses to abiotic and biotic stresses in plants [27]. In this study, unigenes encoding calcium/calmodulins were differentially expressed upon *H. armigera* infestation. In both CT and WT, homologs of calcium-binding proteins, CML29 (XM_020353338.1), CML31(XM_020362287.1), CML11, CML60, PBP1-like (XM_020353210.1) and the calcium uniporter protein (XM_020361379.1) were significantly induced, albeit more in CT than in the WT. In addition, the receptor-like kinase (XM_020346834.1), calcium/calmodulin-regulated receptor–like kinase (XM_020365356.1) and CBL-interacting serine/threonine protein kinase 21 (XM_020371759.1) showed accumulation in WT but were repressed in CT. The endoplasmic reticulum (ER)-associated calcium storage protein calreticulin (XM_020384872.1) showed marked expression in the WT, while the calcium sensor calcineurin B-like protein 4 (XM_020378840.1) had a significant induction in CT. The induction of the calcium/calmodulin-associated proteins in pigeonpea genotypes under the pod borer infestation suggests a role in pest resistance as a second messenger in signal transduction during a herbivory attack (Supplementary Table S3).

### 2.7. Validation of RNA-Seq Analysis by qRT-PCR

**Figure 3:**
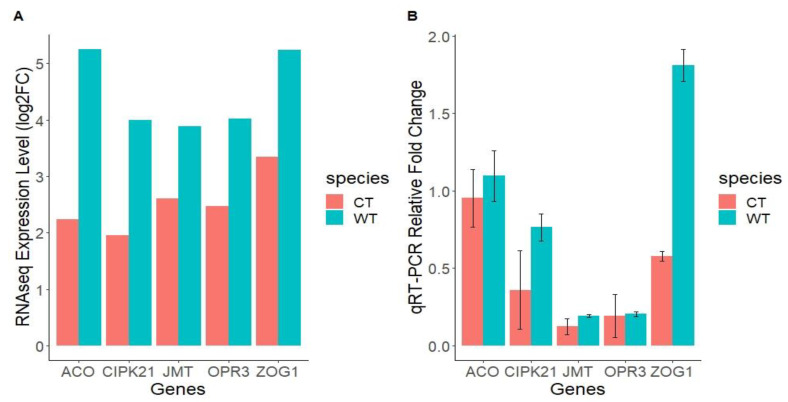
Comparative gene expression trends in cultivated susceptible KAT 60/8 (CT) and resistant wild (WT) genotypes pigeonpea *Cajanus scarabaeoides* accession IBS3471 from (**A**) RNAseq vs. (**B**) Real-time quantitative reverse transcription PCR (qRT-PCR) experiments. ACO: 1-aminocyclopropane-1-carboxylate oxidase, ZOG1: Zeatin O-glucosyltransferase-like, CIPK21: CBL-interacting serine/threonine-protein kinase 21, OPR3: 12-oxophytodienoate reductase 3-like and JMT: Jasmonate O-methyltransferase.

To validate the RNA-Seq results, five genes associated with cytokinin, ethylene, jasmonic acid and calcium-signaling pathways were selected for qRT-PCR validation based on their significant differential expression between the two genotypes. These included two upregulated ethylene pathway genes 1-aminocyclopropane-1-carboxylate oxidase (ACO) and 12-oxophytodienoate reductase 3-like (OPR3), cytokinin pathway zeatin O-glucosyltransferase-like (ZOG1), calcium signaling-associated CBL-interacting serine/threonine-protein kinase 21(CIPK21) and jasmonate O-methyltransferase (JMT) from the jasmonic acid pathway. All the five DEGs exhibited a similar trend between the RNA-Seq and qRT-PCR results (Figure 3A,B), which suggested that our transcriptome analysis was accurate and reliable.

### 2.8. Antinutritional Factors and Herbivore-Induced Plant Volatiles and Metabolites

Most vascular plants emit volatile organic compounds under conditions of abiotic and biotic stresses [28]. Antinutritional factors such as protease inhibitors, phenols and lectin play a role in larval growth reduction, while other organic compounds such as terpenoids provide characteristic smells as insect repellents. In this study, the transcript accumulation of genes encoding secondary metabolites were significantly induced upon infestation in the WT compared to CT. The expression of terpene synthase, an enzyme involved in terpenoids biosynthesis was significantly enhanced in WT, while its expression was suppressed in CT (Table 1). Chalcone synthase (XM_020360142.1), a key enzyme regulating the flavonoid biosynthetic pathway, showed enhanced activity, especially in the WT compared to the CT genotype. A similar trend was observed in other flavonoid and isoflavonoid pathway enzymes that were suppressed in CT and their expression enhanced in the WT. For example, isoflavone-7-O-methyltransferase 6-like (XM_020354442.1), dihydroflavonol 4-reductase-like (XM_020375855.1), flavonoid 3’,5’-hydroxylase 2-like XM_020381106.1) and anthocyanidin reductase (XM_020383227.1) expression was highly suppressed in CT, while the expression of flavonol synthase (XM_020366267.1), flavin-containing monooxygenase (XM_020372407.1) and isoflavone 4’-O-methyltransferase-like (XM_020381575.1) was enhanced in the WT genotype (Supplementary Table S3). The transcripts of phenylalanine ammonia-lyase (XM_020374082.1), an enzyme involved in the first step in phenylpropanoid biosynthesis organic compounds involved in several biological functions in plants, including defense against herbivores and pathogens, showed a marked expression in the WT. Other significantly induced transcripts in the WT included Kunitz trypsin inhibitor 2-like (XM_020357544.1) and nerolidol synthase 1-like (XM_020383219.1) (Table 1).

### 2.9. Gene Ontology Analysis Revealed Genes Enriched in Signaling and Defense-Related Pathways

**Figure 4:**
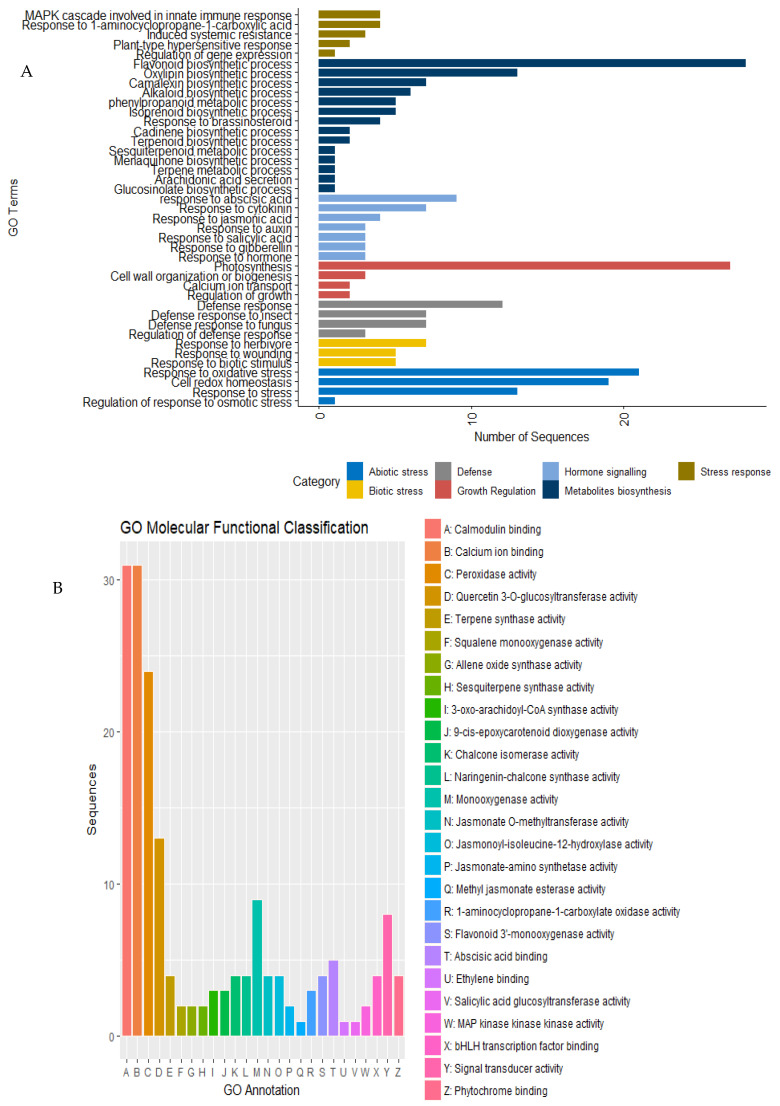
Gene ontology enrichment showing (**A**) the biological processes and (**B**) molecular functions associated with the differentially expressed genes in both cultivated and tolerant wild pigeonpea genotypes.

To have a better understanding of the biological processes associated with significantly expressed genes and their functional roles in plant defense against insect herbivory, we performed a Gene Ontology (GO) enrichment analysis to identify over-represented terms for the DEGs using Blast2GO [29]. In both CT and WT, the DEGs were linked to biological processes associated with response to wounding, jasmonic acid, abscisic acid, salicylic acid, defense response, oxidative reduction, the regulation of transcription and photosynthesis, among others, while the molecular activities included protein binding, the transcription factor, serine/threonine kinase, calmodulin, calcium ion binding and unfolded protein binding (Figure 4A). However, the WT genotype was associated with additional biological processes, including the response to stress, systemic acquired resistance, isoprenoid biosynthesis, response to brassinosteroid, arachidonic acid secretion, glycerolipid biosynthesis, induced systemic resistance, jasmonic acid-mediated signaling, the positive regulation of transcription and glucosinolate biosynthesis. The molecular activities associated with the WT genotype were terpene synthase, transmembrane transporter, enzyme inhibitors, sulfoquinovosyltransferase, peroxidase, ammonia-lyase, phospholipase A2 and chitinase (Figure 4B).

## 3. Discussion

The resistance to stress varies among individual species as a result of differences in their genetic makeups. Differences in natural selection and artificial selection processes during domestication account for the presence of populations with varied levels of resistance, ranging from susceptible to resistant towards specific pest species [30,31]. Compared to domesticated cultivars, crop wild relatives (CWRs) have been challenged in severer natural environments for thousands of years and maintain a much higher level of genetic diversity. CWRs possess sophisticated defense mechanisms compared to their cultivated counterparts attributed to their long-term exposure to varied stress factors [13,32]. Thus, CWRs could hold great potential as reservoirs for novel genetic resources for crop biotic and abiotic stress improvements.

In this study, the investigation of the genotype-specific response to *H. armigera* damage in *Cajanus scarabaeoides*, a pest-resistant pigeonpea wild relative (WT), and KAT60/8, a susceptible cultivated (CT) genotype, was conducted. Extensive feeding was observed in the susceptible CT genotype, with minimal feeding in the tolerant WT. Similar results were observed when ICPL87, the insect susceptible check, and *C. scarabaeoides* accession IBS3471 were subjected to the *H. armiger* a bioassay. Under the no choice petri plate assay, limited feeding was observed in WT, suggesting the presence of resistance mechanisms against defoliation (Figure 1A,B). Using high-throughput sequencing and an in-depth transcriptomics analysis, herbivore-induced genes that could be involved in defense responses against *H. armigera* infestation were identified. It was apparent that many transcripts were responsive to pod borer feeding in both CT and WT, but more genes were differentially expressed in CT compared to the WT (Figure 2B). The lower number of expressed unigenes observed in the WT could be attributed to the low reads mapping rate against the pigeonpea genome (Figure 1E). Although *C. scarabaeoides* is a wild relative of the cultivated *C. cajan*, the low mapping rate suggests some unigenes could have been missed. Only 15% of the DEGs were common between the two accessions, suggesting unique molecular responses eliciting different pathways (Figure 2C). Quantitative real-time polymerase chain reaction (qRT-PCR) gene expression validation with ICPL87, a well-known *H. armigera* susceptible pigeonpea cultivar, and a resistant wild pigeonpea *Cajanus scarabaeoides* accession IBS3471 showed similar expression trends with CT and WT RNA-Seq results (Figure 3A,B). These results suggest that the response to insect challenges in terms of gene expression trends were similar in susceptible cultivated pigeonpea genotypes KAT 60/8 (CT) and cultivar ICPL87.

The results indicate a network of complex and coordinated signaling involving phytohormone signaling, transcriptional regulation and the biosynthesis of metabolites and volatiles as a resistance strategy. The plant hormones JA, SA and ET play important roles in modulating the plant defense against various diseases and pests [33]. Three important enzymes in the JA biosynthesis pathway: lipoxygenase, oxophytodienoate reductase and allene oxide synthase, showed enhanced accumulation in WT compared to CT (Table 1 and Figure 3). JA plays a key role activating a defense against biotic attacks, including chewing insects [18]. Many wound-induced adaptive responses are triggered by JA through autonomous and nonautonomous pathways [34]. The ET pathway enzymes 2-oxoglutarate (2OGD), 1-aminocyclopropane-1-carboxylate synthase and 1-aminocyclopropane-1-carboxylate oxidase expression were enhanced in the WT upon herbivory (Supplementary Table S3). Several studies have shown the synergistic operation of JA and ET signaling in the activation of defense-related genes. The ethylene responsive factor 1 (ERF 1) was reported as a positive regulator of JA and ET signaling, and several members of the (ERF) family play an important role in mediating the defense responses in Arabidopsis [35,36].

Calcium (Ca^2+^) has long been considered a crucial component in the wound-signaling pathway [37]. Calcium is a universal second messenger activated early in signaling cascades in plants as a mediator in response to a wide array of abiotic and biotic stimuli [38]. Plants’ responses to herbivory include the reaction to both mechanical wounding and elicitors from insect oral secretions. The significant induction of several Ca^2+^-binding proteins in both CT and WT genotypes (Table 1 and Supplementary Table S3) suggest an enhanced cellular Ca^2+^ concentration upon a *H. armigera* attack reaching a threshold to bind and trigger the functions of calmodulin-binding proteins [39]. In Arabidopsis, *Spodoptera littoralis* oral secretions activated the systemic defense through the induction of cytosolic Ca^2+^ and JA phytohormone elevations, resulting in the upregulation of CML42, a crucial signaling component connecting Ca^2+^ and JA signaling in response to biotic stress [40]. Ca^2+^-dependent protein kinases can phosphorylate WRKY TFs, leading to direct the post-translational regulation of the TF activities [41]. It has been demonstrated that the different Ca^2+^ amplitudes might be involved in the coordination of several signaling branches during the defense response [42]. Ca^2+^/calmodulin-mediated regulation could therefore function in orchestrating a complex interplay between regulatory pathways to establish a resistance response against a herbivory attack.

Plants respond actively to insect herbivory and mechanical wounding by inducing distinctive biochemical changes that involve the production of phytohormones, secondary metabolites and proteins with antimicrobial (phytoalexins), insecticidal, antioxidant activity and other secondary metabolites at the site of injury or systematically in distant unwounded tissues [43]. Several biosynthetic pathways, including flavonoid, phenylpropanoid and stilbenoid, are important in plant defense. Chalcone synthase (CHS), a key enzyme in the flavonoid and isoflavonoid biosynthesis pathway was highly induced in the WT in response to the *H. armigera* infestation (Table 1). Flavonoids play an important role in plant defense, and CHS acts as the gatekeeper in flavonoid biosynthesis pathway regulation [41]. The enhanced production of CHS in the WT (Table 1) suggests a unique role of CHS in flavonoids production in response to plant biotic stress [44]. The accumulation of flavonoids as a result of CHS activity negatively regulates auxin transport [45], which was found to increase the resistance to *Fusarium oxysporum* in tomatoes [46]. Upon herbivory, higher levels of Kunitz trypsin inhibitor 2 (KTI2) and Nerolidol synthase 1 expression were observed in WT compared to CT (Table 1). Trypsin inhibitors are herbivore and wound-induced secondary metabolites that inhibit insect digestive proteases in the larval midgut, leading to reduced insect growth rates or increase mortality [47]. Nerolidol synthase 1 (NSE1) is associated with the biosynthesis of monoterpene, a volatile compound that is toxic to insects [48,49]. The transcript levels of both KTI and NSE1 accumulated to high levels in the resistant WT genotype, suggesting their contribution to the defense response against *H. armigera*.

Several TFs had disparate expressions in both the CT and WT in response to insect herbivory. the expression of seven WRKY TFs was induced in the WT compared to CT. WRKY TFs are one of the largest family of regulatory proteins involved in the insect pest and pathogen defense response [50,51]. Two insect-responsive WRKY genes: WRKY3, whose transcripts accumulate in response to wounding, and WRKY6, whose wound responses are significantly amplified when fatty acid–amino acid conjugates (FACs) in larval oral secretions are introduced into wounds during feeding have been reported in *Nicotiana attenuate* [52]. The response to wounding and herbivore-specific signals (FACs) demonstrates that WRKYs help plants to differentiate mechanical wounding from herbivore attacks, mediating herbivore-specific defenses. bHLH, MYC2, MYC3 and MYC4 have additive control of the JA-dependent responses, including root growth inhibition, the bacterial pathogen defense and the defense against insect herbivory [53]. In Arabidopsis, NAC TFs play an important role in the regulation of plant defense responses against different pathogens, in addition to wounding and insect feeding [54].

## 4. Materials and Methods

### 4.1. Plant Materials

Pod borer susceptible cultivated *Cajanus cajan* KAT60/8 (CT) genotype and *Cajanus scarabaeoides* (WT) resistant wild relative accession used in this study were obtained from Kenya Agriculture and Livestock Research Organization (KALRO) Katumani, Kenya and International Livestock Research Institute (ILRI) Forage Genebank, Ethiopia, respectively. The insect susceptible check pigeonpea cultivar ICPL87 and the resistant wild pigeonpea *Cajanus scarabaeoides* accession IBS3471 kindly provided by the Australian Grains Genebank were used in the qRT-PCR analysis. The seeds were scarified, surface-sterilized with 1% sodium hypochlorite and germinated on petri plates in the laboratory. Young seedlings were transplanted into plastic pots and maintained under natural daylight in a screen house for four weeks. The plants were transferred into an environment chamber with the temperature set at 27 °C, 70% relative humidity and a 16h:8h photoperiod for the insect bioassay experiment.

### 4.2. Insect Bioassay

The *Helicoverpa armigera* larvae used for the bioassay at BecA-ILRI’s labs were kindly provided by Kenya Biologics Ltd. (Kenya Biologics Ltd., Thika, Kenya), and *H. armigera* larvae used for experiments at the Centre for Agriculture and the Bioeconomy (CAB), Queensland University of Technology were hatched from eggs obtained from the Commonwealth Scientific and Industrial Research Organization (CSIRO), (Narrabri, NSW, Australia). The larvae were maintained in an environment chamber at 25 °C, a 12 h:12 h photoperiod and 60% relative humidity before the bioassay experiment. The plants were divided into treatment and control sets comprising six pots per genotype. Two larvae (at the third instar stage) were introduced to each pot containing plants with fully expanded leaves enclosed in cage nets to restrict the movements of larvae to other plants for 24 h. The control group comprising plants in six pots were maintained under similar conditions but without the introduction of *H. armigera* larvae. Triplicate leaf samples from *H. armigera*-infested and control plants were collected at 0 h and 24 h after insect infestation, snap-frozen in liquid nitrogen and stored at −80 °C for RNA isolation. For detached leaf bioassay, leaves from both CT and WT were sampled in triplicate in a 90 mm petri plate. A single larvae per plate were introduced onto the samples, and feeding was assessed after 24 h.

### 4.3. Total RNA Extraction and High-Throughput Sequencing

Total RNA was extracted from 12 leaf tissue samples from CT and WT genotypes using the RNeasy plant mini kit (Qiagen) according to the manufacturer’s instructions. Briefly, approximately 100 mg of plant leaf tissue was ground using pestle and mortar in liquid nitrogen and transferred into 2 mL Eppendorf tubes. The lysate was homogenized in a QIAshredder spin column, and 450 µL lysis buffer was added and vortexed and centrifuged at maximum speed for 2 min. The flowthrough supernatant was transferred into new tubes, and half-volume ethanol was added and mixed by pipetting. Approximately 650 µL of the sample was transferred to a RNeasy spin column and centrifuged for 15 s, and the flowthrough was discarded. A 700-µL RW1 buffer was added, centrifuged for 15 min and the flowthrough discarded. A 500 µL of RPE buffer was twice added, centrifuged at 15 s and 2 min, respectively, and the flowthrough was discarded. Total RNA was eluted with 50 µL of RNase-free water and stored at −80 °C for downstream analysis. Total RNA quantity and purity was checked with a NanoDrop^®^ 2000 spectrophotometer (Thermo Fisher Scientific, Fair Lawn, NJ, USA). Four triplicate RNA libraries were prepared using the TruSeq^®^ Stranded Total RNA with a Ribo-Zero Plant kit (Illumina, San Diego, CA, USA) using the manufacturer’s instructions. The library integrity and quality were verified using 2% agarose gel electrophoresis and TapeStation (Agilent Technologies, Santa Clara, CA, USA) and 151-bp paired-end reads sequenced using an Illumina^®^ Mi-Seq platform at BecA-ILRI Hub, Kenya. The raw data were deposited in the NCBI Short Read Archive (SRA) database under Accession Number PRJNA630454.

### 4.4. Gene Expression Validation with Real-Time Quantitative Reverse Transcription PCR (qRT-PCR)

The expression validation was conducted using ICPL87, a well-known *H. armigera* susceptible pigeonpea cultivar and a resistant wild pigeonpea *Cajanus scarabaeoides* accession IBS3471 at the CAB lab (Queensland University of Technology, Brisbane, Australia). The analysis was conducted on both ICPL87 and IBS3471 at 0 and 24 h after the insect bioassay. Two-third instar larvae were introduced to each plant and allowed to feed for 24 h. Triplicate leaf samples from *H. armigera*-infested and control plants were collected at 0 h and 24 h after insect infestation, snap-frozen in liquid nitrogen and stored at −80 °C for RNA isolation. Total RNA was extracted from leaf samples using a RNeasy^®^ Plant mini kit following the manufacturer’s protocols. cDNA synthesis was carried out using M-MLV Reverse-Transcriptase (Promega M170A) according to the manufacturer’s protocols. Gene-specific primers were designed from selected DEGs from both genotypes using Primer3 software [55]. The template cDNAs were diluted to 25-fold, and 2 μL was added to the qRT-PCR reaction mixture (10 μL) made of 5 μL of GoTaq^®^ qPCR Master Mix, 0.3 μL of each primer (10 μM) and 2.4 μL of nuclease-free water. The qRT-PCR was conducted on a Bio-Rad C1000 Touch™ Thermal Cycler (Model CFX384™ Real-Time System, Singapore) with the thermal cycle conditions: 95 °C for 3 min, 45 cycles at 95 °C for 10 s and 60 °C for 30 s and a melting profile at 95 °C for 10 s, 65 °C for 5 s and 95 °C for 5 s. Each sample had three biological and three technical replicates for each time point. The relative fold change was calculated using the 2^−ΔΔCt^ method [56] and the plots generated using the ggplot2 R package (Figure 3). *Cajanus cajan* actin 11 and initiation factor 4α (IF4α) were used as the reference genes. All the primer sequences are listed in Supplementary Table S4.

### 4.5. Differential Expression and GO Enrichment Analysis

Raw reads were preprocessed for quality check and the removal of adapters and low-quality reads. Quality of reads was assessed by the FastQC program (version 0.11.5, http://www.bioinformatics.babraham.ac.uk/projects/fastqc/) and trimmed by Trimmomatic (version 0.38, http://www.usadellab.org/cms/index.php?page=trimmomatic) [57]. High-quality reads were mapped against the pigeonpea genome (C. cajan_V1.0 GCF_000340665.1) [58] using the Tophat program (ver. 2.1.0, https://ccb.jhu.edu/software/tophat/index.shtml). Cufflinks and ht-Seq count were used to estimate the gene expression levels through comparison of the pest-infested reads at 24 h against the 0 h controls to generate the expressed transcript counts. Differentially expressed genes were determined using the DESeq2 R package (https://bioconductor.org/packages/release/bioc/html/DESeq2.html). Only those genes with an expression of more than 2-fold change, a *p*-value < 0.001 and a false discovery rate (FDR) of <0.05 were considered differentially expressed. A heatmap of differentially expressed genes was generated in the shinyHeatmaply R package (https://cran.r-project.org/web/packages/shinyHeatmaply/index.html). GO enrichment analysis of the represented GO terms in the genotypes was conducted using Blast2GO [59] with FDR < 0.05 using the sequences of the differentially expressed transcripts in the two cultivars.

## 5. Conclusions

The use of pest-resistant crop varieties is an important component of the integrated pest management system. This practice could not only provide high economic benefits but, also, offer high social benefits by protecting plants against their natural pests in an environmentally friendly manner. The availability of crops’ wild relatives provides a gene pool that could be explored to fight against the abiotic and biotic constraints limiting the productivity of agricultural crops. Although the pigeonpea wild relative comprises poor agronomic characteristics ranging from poor stature to small seed size, they hold important pest and disease resistance traits. Our findings in this study will enhance the understanding of the response to insect damage in the *H. armigera*-resistant pigeonpea wild relatives and provide a foundation for the further elucidation of specific candidate gene(s) that could be utilized in pigeonpea improvement against pod borers.

## Figures and Tables

**Table 1:** Comparison of selected herbivory-induced genes and pathways shared between susceptible cultivated genotype (CT) and resistant wild accession (WT).

Susceptible Cultivated (CT)			Tolerant Wild (WT)			
Accession	Log_2_FC	*p*-Value	Log_2_FC	*p*-Value	Description	Pathway
XM_020361379.1	3.31	4.12 × 10^−7^	3.86	3.81 × 10^−11^	calcium uniporter protein 2	Ca^2+^ signalling
XM_020383212.1	−5.45	1.35 × 10^−4^	2.26	2.72 × 10^−3^	calmodulin receptor-like kinase	Ca^2+^ signalling
XM_020371759.2	1.96	1.00 × 10^−5^	6.92	8.29 × 10^−18^	CBL-interacting serine/threonine-protein kinase 21	Ca^2+^ signalling
XM_020370652.1	2.47	6.74 × 10^−8^	4.02	3.08 × 10^−13^	12-oxophytodienoate reductase	Ethylene
XM_020349666.1	8.11	3.13 × 10^−18^	9.30	3.74 × 10^−25^	ABR1-like	Ethylene
XM_020353227.2	2.29	3.76 × 10^−9^	3.81	1.26 × 10^−10^	1-aminocyclopropane-1-carboxylate oxidase	Ethylene
XM_020357438.2	3.34	4.21 × 10^−4^	5.24	0.000485	zeatin O-glucosyltransferase-like	Cytokinin
XM_020370529.1	−3.56	2.44 × 10^−9^	6.86	5.86 × 10^−9^	chalcone synthase 1	Flavonoid
XM_020357544.1	6.88	1.25 × 10^−9^	10.90	5.89 × 10^−36^	kunitz trypsin inhibitor	Inhibitors
XM_020364128.1	3.22	3.17 × 10^−7^	7.18	1.21 × 10^−10^	wound-induced trypsin inhibitor	Inhibitors
XM_020376230.1	−1.27	3.23 × 10^−3^	6.19	3.60 × 10^−6^	2-hydroxyisoflavanone synthase	Isoflavonoid
XM_020384234.1	5.57	1.11 × 10^−4^	8.95	8.29 × 10^−18^	isoflavone-7-O-methyltransferase	Isoflavonoid
XM_020377202.1	−2.60	3.34 × 10^−5^	10.14	3.38 × 10^−37^	2-oxoglutarate-dependent dioxygenase	Jasmonic acid
XM_020350219.1	2.76	5.51 × 10^−10^	4.52	2.48 × 10^−9^	allene oxide synthase	Jasmonic acid
XM_020355903.1	−2.12	3.28 × 10^−7^	7.13	6.50 × 10^−19^	linoleate 9S-lipoxygenase	Jasmonic acid
XM_020365833.2	2.61	4.25 × 10^−4^	3.89	0.000155	jasmonate O-methyltransferase	Jasmonic acid
XM_020383219.1	2.68	2.16 × 10^−4^	10.12	1.02 × 10^−29^	nerolidol synthase 1	Terpene
XM_020353681.1	−4.46	2.51 × 10^−6^	8.71	5.98 × 10^−14^	terpene synthase 2	Terpene
XM_020383551.1	5.62	5.20 × 10^−11^	5.59	1.75 × 10^−4^	bHLH18	Transcription factor
XM_020382733.1	−1.79	1.97 × 10^−3^	5.47	1.37 × 10^−4^	bZIP11	Transcription factor
XM_020383974.1	4.52	4.92 × 10^−9^	5.72	5.05 × 10^−5^	ERF RAP2	Transcription factor
XM_020371421.1	−2.72	9.95 × 10^−4^	5.70	5.97 × 10^−4^	MYB4	Transcription factor
XM_020351385.1	1.13	1.51 × 10^−3^	5.72	7.70 × 10^−11^	MYC4	Transcription factor
XM_020349871.1	4.36	4.93 × 10^−3^	2.59	1.47 × 10^−9^	MYC2	Transcription factor
XM_020376674.1	3.88	3.03 × 10^−4^	2.57	8.38 × 10^−4^	WRKY 41	Transcription factor
XM_020374599.1	2.06	1.89 × 10^−4^	3.00	6.17 × 10^−5^	WRKY 51	Transcription factor

## Data Availability

The data generated in this study was deposited in the NCBI Short Read Archive (SRA) database under Accession Number PRJNA630454.

## Supplementary Materials

Supplementary Materials can be found at https://www.mdpi.com/1422-0067/22/1/309/s1. Table S1: List of differentially expressed genes in the cultivated susceptible (CT) pigeonpea genotype; Table S2: List of differentially expressed genes in the tolerant wild (WT) pigeonpea genotype; Table S3: List of differentially expressed genes in both CT and WT genotypes; Table S4: List of primer sequences used in the qRT-PCR expression analysis.

## Abbreviations

TF	Transcription factor
ET	Ethylene
JA	Jasmonic acid
DEG	Differentially Expressed Genes
CPM	Counts per million
ERF	Ethylene-responsive factor
CHS	Chalcone synthase
CWRs	Crop Wild Relatives
