# Supplementary material for: Comparative Analysis Delineates the Transcriptional Resistance Mechanisms for Pod Borer Resistance in the Pigeonpea Wild Relative Cajanus scarabaeoides (L.) Thouars
Source: Int J Mol Sci. 2020 Dec 30;22(1):309. doi: 10.3390/ijms22010309 (PMC7795875; doi:10.3390/ijms22010309)
Supplement: Supplementary file 1 [file ijms-22-00309-s001.zip › Supplementary_files/Supplementary Table S4.docx]

Supplemetary Table S4: List of primers used in the study for qRT-PCR expression analysis

| **Gene ID** | **Primer sequence 5' - 3'** | **Amplicon size (bp)** | **Source** |
| --- | --- | --- | --- |
| Zeatin O-glucosyltransferase (ZOG1) | F- ATGCACTCTGACCAACCCAG | 133 | This study |
|  | R- TCAACCTCCTCACGGCATTC |  |  |
| 1-aminocyclopropane-1-carboxylate oxidase (ACO) | F- GACACCGTGGAGAGGATGAC | 121 |  |
|  | R- CCCAGTCCATGTCCTTGACC |  |  |
| CBL-interacting serine/threonine-protein kinase 21 (CIPK21) | F- GCCGCTGCAACAGATGTAAG | 131 |  |
|  | R- GCAACTTCAATCACCTGGGC |  |  |
| 12-oxophytodienoate reductase 3 (OPR3) | F- TGCCATTGACTCTGACCCAC | 134 |  |
|  | R- CGGTTTGGCCATAAGCTGTG |  |  |
| Jasmonate O-methyltransferase (JMT) | F- TCGATTGGGACGGTGGAATG | 82 |  |
|  | R- GCCCTAATGGTCCTAGCCAC |  |  |
| Initiation factor 4α | F- GCCGAGATCACACAGTCTCA | 95 | Sinha et al., 2015 |
|  | R- ACCACGAGCCAAAAGATCAG |  |  |
| Actin 11 | F- GGCATACATTGCCCTTGACT | 97 |  |
|  | R- GAACCTCGGGACATCTGAAA |  |  |
